# Lung stiffness of C57BL/6 *versus* BALB/c mice

**DOI:** 10.1038/s41598-023-44797-x

**Published:** 2023-10-14

**Authors:** Andrés Rojas-Ruiz, Magali Boucher, Rebecka Gill, Louis Gélinas, Fun-Qun Tom, Liah Fereydoonzad, Percival Graham, Jorge Soliz, Ynuk Bossé

**Affiliations:** 1 Institut Universitaire de Cardiologie et de Pneumologie de Québec (IUCPQ)-Université Laval, Pavillon A, room 2089, 2725, chemin Sainte-Foy, Quebec, QC G1V 4G5 Canada; 2 SCIREQ Inc., Montreal, QC Canada

**Keywords:** Physiology, Systems biology, Medical research

## Abstract

This study was undertaken to determine whether a smaller lung volume or a stiffer lung tissue accounts for the greater lung elastance of C57BL/6 than BALB/c mice. The mechanical properties of the respiratory system and lung volumes were measured with the flexiVent and compared between male C57BL/6 and BALB/c mice (n = 9). The size of the excised lung was also measured by volume liquid displacement. One lobe was then subjected to sinusoidal strains in vitro to directly assess the mechanical properties of the lung tissue, and another one was used to quantify the content of hydroxyproline. In vivo elastance was markedly greater in C57BL/6 than BALB/c mice based on 5 different readouts. For example, respiratory system elastance was 24.5 ± 1.7 *vs.* 21.5 ± 2.4 cmH_2_O/mL in C57BL/6 and BALB/c mice, respectively (p = 0.007). This was not due to a different lung volume measured by displaced liquid volume. On the isolated lobes, both elastance and the hydroxyproline content were significantly greater in C57BL/6 than BALB/c mice. These results suggest that the lung elastance of C57BL/6 mice is greater than BALB/c mice not because of a smaller lung volume but because of a stiffer lung tissue due to a greater content of collagen.

## Introduction

C57BL/6 and BALB/c mice are the most widely employed mouse strains in experimental lung research, in particular for modeling human respiratory diseases^1,2^. Several respiratory diseases such as interstitial lung disease (ILD) and chronic obstructive pulmonary disease (COPD) are characterized by a change in lung elastance^3–6^. It is also well known that aberrant lung elastance alters lung function^7^. For example, both ILD and COPD, characterized with higher and lower than normal elastance, respectively, lead to a decline in the spirometric measurement of the forced expiratory volume in 1 s (FEV_1_).

Interestingly, the elastance of the respiratory system is markedly greater in C57BL/6 than BALB/c mice^8–14^. The increased elastance of C57BL/6 *versus* BALB/c mice is also observed in open-chest conditions (*i.e.*, following bilateral thoracotomies)^13^, suggesting that lung elastance, and not necessarily the elastance of the chest wall, is contributing to the different elastance of the respiratory system between these two mouse strains. The underlying reasons for the increased lung elastance of C57BL/6 *versus* BALB/c mice are undefined. The stiffness of the lung tissue, defined by the amount of stress required to strain the tissue, is obviously an important determinant of lung elastance. Yet, lung volume is another main determinant. This can be epitomized by comparing elastance and volume between the lung of humans and mice. The elastance of a human lung (~ 0.005 cmH_2_O/mL), is about 3.7 logs inferior to a mouse lung (~ 30 cmH_2_O/mL)^8,15^, mainly because it is also about 3.7 logs bigger (6000 *vs.* 1 mL).

Whether C57BL/6 mice have a greater lung elastance than BALB/c mice because of a smaller lung volume, a stiffer lung tissue, or a combination thereof, is unknown. Untangling the determinants of this conspicuous strain difference may hint investigators on the most suitable mouse strains to choose from for addressing key questions in specific models of human respiratory diseases. This study was undertaken to determine whether a smaller lung volume or a stiffer lung tissue accounts for the greater lung elastance of C57BL/6 than BALB/c mice.

## Results

**Figure 1:**
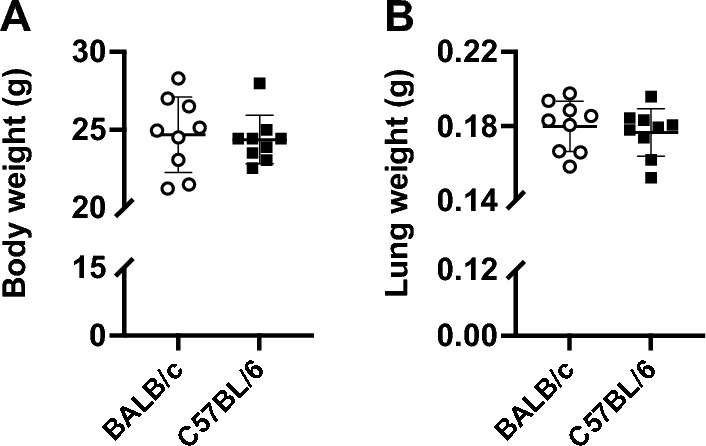
Body weight of mice (**A**) and total lung wet weight (**B**) are shown for BALB/c (open circles) and C57BL/6 (solid squares) mice. Data are individual results, together with means ± SD. N = 9 per group.

**Figure 2:**
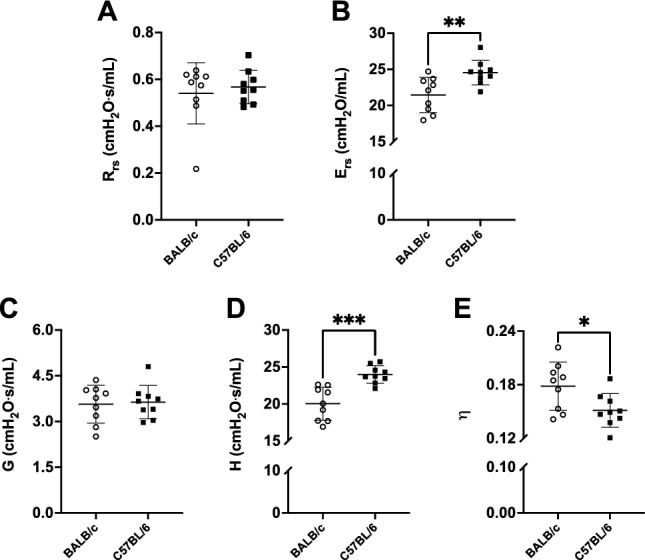
Respiratory mechanics measured through oscillometry by the flexiVent. Respiratory system resistance (R_rs_) (**A**), respiratory system elastance (E_rs_) (**B**), tissue resistance (G) (**C**), tissue elastance (H) (**D**) and hysteresivity (η) (**E**) are shown for BALB/c (open circles) and C57BL/6 (solid squares) mice. Data are individual results, together with means ± SD. Significant differences are indicated by asterisks (*, ** and *** p < 0.05, 0.01 and 0.001 respectively). N = 9 per group.

The mouse weight was not different between C57BL/6 (24.4 ± 1.5 g) and BALB/c (24.7 ± 2.4 g) mice (Fig. 1A). The wet lung weight was also identical between strains (Fig. 1B). However, respiratory mechanics, assessed through oscillometry, was significantly different between the two strains (Fig. 2). In particular, respiratory system elastance (E_rs_) was higher in C57BL/6 than BALB/c mice (24.54 ± 1.70 *vs.* 21.45 ± 2.43 cmH_2_O/mL, p = 0.007) (Fig. 2B). Similarly, tissue elastance (H) was higher in C57BL/6 than BALB/c mice (23.99 ± 1.18 *vs.* 20.03 ± 2.24 cmH_2_O s/mL, p = 0.0002) (Fig. 2D). Since tissue resistance (G) was not different between mouse strains (Fig. 2C), the increased H resulted in a lower hysteresivity (η) in C57BL/6 than BALB/c mice (0.18 ± 0.03 *vs.* 0.15 ± 0.02, p = 0.02) (Fig. 2E).

**Figure 3:**
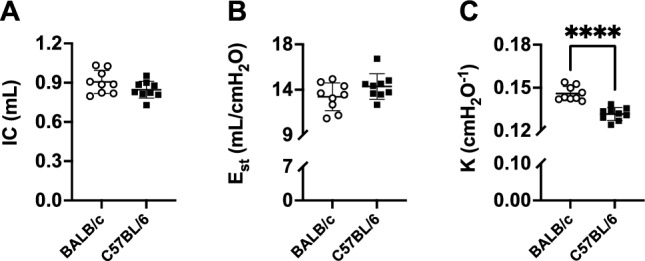
FlexiVent readouts from the deep inflation (DI) maneuver and the stepwise, pressure-controlled, partial pressure–volume (P–V) maneuver. Inspiratory capacity (IC) (**A**), quasi-static elastance (E_st_) (**B**) and the parameter K of Salazar-Knowles equation (**C**) are shown for BALB/c (open circles) and C57BL/6 (solid squares) mice. Data are individual results, together with means ± SD. Significant differences are indicated by asterisks (**** is p < 0.0001). N = 9 per group.

Inspiratory capacity (IC), assessed from the deep inflation (DI) maneuver, was not different between the two mouse strains (Fig. 3A). The quasi-static elastance (E_st_), assessed from the stepwise, pressure-controlled, partial pressure–volume (P–V) maneuver, was numerically higher in C57BL/6 than BALB/c mice, but this difference was not significant (p = 0.12) (Fig. 3B). However, the parameter K of Salazar-Knowles equation, which is a volume-independent indicator of lung compliance, was clearly lower in C57BL/6 *versus* BALB/c mice (0.132 ± 0.004 *vs.* 0.146 ± 0.005/cmH_2_O, p ˂ 0.0001) (Fig. 3C).

**Figure 4:**
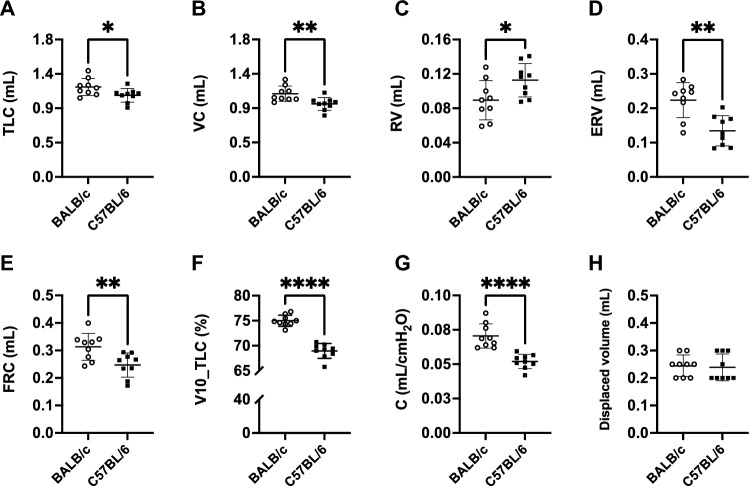
FlexiVent readouts from the ramp-style, full-range, pressure–volume (P–V) maneuver and the excised lung volume measured by liquid displacement. Total lung capacity (TLC) (**A**), vital capacity (VC) (**B**), residual volume (RV) (**C**), expiratory reserve volume (ERV) (**D**), functional residual capacity (FRC) (**E**), lung volume at 10 cmH_2_O expressed in percentage of TLC (V10_TLC) (**F**), lung compliance (**G**) and excised lung volume (**H**) are shown for BALB/c (open circles) and C57BL/6 (solid squares) mice. Data are individual results, together with means ± SD. Significant differences are indicated by asterisks (*, ** and **** are p < 0.05, 0.01 and 0.0001, respectively). N = 9 per group.

Many lung volumes, assessed from the dynamic, ramp-style, full-range P–V maneuver, were significantly lower in C57BL/6 than BALB/c mice (Fig. 4). These included total lung capacity (TLC) (p = 0.03), vital capacity (VC) (p = 0.007), expiratory reserve volume (ERV) (p = 0.001) and functional residual capacity (FRC) (p = 0.008) (Fig. 4A, B, D and E respectively). Contrastingly, residual volume (RV) was higher in C57BL/6 compared to BALB/c mice (0.11 ± 0.02 *vs.* 0.09 ± 0.02 mL, p = 0.03) (Fig. 4C). Furthermore, lung compliance was lower in C57BL/6 than BALB/c mice (p ˂ 0.0001) (Fig. 4F and G). There was no difference in total lung volume between the two mouse strains when the lung was excised and measured at zero transpulmonary pressure by liquid displacement (Fig. 4H).

**Figure 5:**
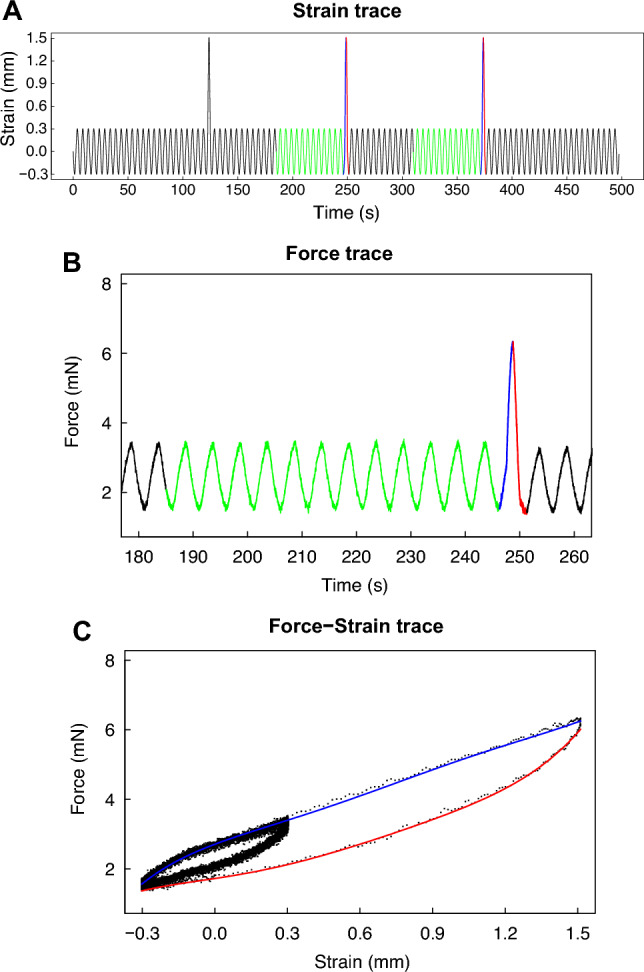
Protocol to measure lung tissue mechanics of the right inferior lobe in vitro. Each lobe was subjected to 3 consecutive sequences of sinusoidal small-amplitude strain oscillations for 2 min followed by a half-sine stretch of 30%. A representative strain trace is shown in (**A**). The green section prior to the second and the third 30% stretches are locations where the mechanical properties of the lobe (*i.e.*, elastance, resistance and hysteresivity) were calculated during the small-amplitude oscillations. They were selected to be away (*i.e.*, 1 min) from the large 30% stretch, where the mechanical properties had nearly reached a steady-state. The blue and red sections are locations of the second and third half-sine stretches of 30%, where the mechanical properties of the lobe were calculated during the large-amplitude oscillations. They were selected because they were the first 30% stretches where the mechanical properties of the lobe had reached a near steady-state. The corresponding force trace before, during, and after the second 30% stretch is shown in (**B**). The corresponding force-strain trace is shown in (**C**). The blue and red lines are actual data fits during the 30% stretch and retraction, respectively. Together, they were used to calculate hysteresis (*i.e.*, the area within the loop), which was then used to sequentially calculate resistance, hysteresivity and elastance. The traces in (**A**) and (**B**) look continuous but are, in fact, constituted of discrete data points sampled at 100 Hz, as can be seen in (**C**).

**Figure 6:**
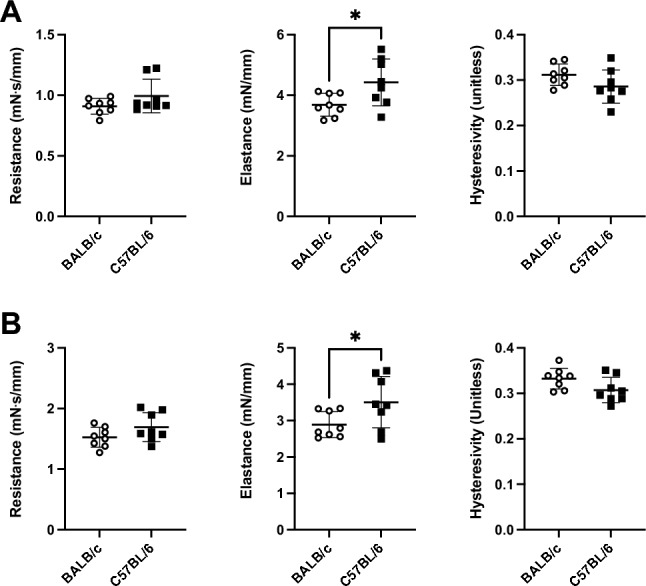
Lung tissue mechanics measured in vitro on the right inferior lobe. Resistance, elastance and hysteresivity measured during either small-amplitude oscillations (**A**) or large-amplitude oscillation (**B**) are shown for BALB/c (open circles) and C57BL/6 (solid squares) mice. Data are individual results, together with means ± SD. Significant differences are indicated by asterisks (* is p < 0.05). N = 8 per group.

Lung tissue mechanics, measured in vitro by subjecting the right inferior lobe to sinusoidal strains of either small or large amplitudes (Fig. 5), are depicted in Fig. 6. The length of the right inferior lobe was not different between C57BL/6 and BALB/c mice (4.6 ± 0.5 *vs.* 4.9 ± 0.4 mm; p = 0.28). Since the weight of the mice, as well as the volume and the weight of the whole lung were not different between the two mouse strains, it was assumed that the cross-sectional area of the right inferior lobe was also comparable between mouse strains. Elastance was significantly higher in C57BL/6 *versus* BALB/c lobes, irrespective of whether it was tested during small-amplitude oscillations (4.43 ± 0.77 *vs.* 3.67 ± 0.38 mN/mm, p = 0.03) (Fig. 6A) or large-amplitude oscillations (3.51 ± 0.71 *vs.* 2.89 ± 0.36 mN/mm, p = 0.04) (Fig. 6B). There were no significant differences in lobe tissue resistance and hysteresivity between the two mouse strains. All physiological results are summarized in Table 1.

**Table 1:** Summary of results.

Readouts	Abbrv.	Mean ± SD		Units	P value
		BALB/c	C57BL/6		
Mouse weight	–	24.70 ± 2.41	24.38 ± 1.55	g	0.741
Wet lung weight	–	0.18 ± 0.01	0.18 ± 0.01	g	0.602
Respiratory system resistance	R_rs_	0.54 ± 0.13	0.57 ± 0.07	cmH_2_O s/mL	0.592
Respiratory system elastance	E_rs_	21.45 ± 2.43	24.54 ± 1.70	cmH_2_O/mL	**0.007**
Newtonian resistance	R_N_	0.26 ± 0.08	0.29 ± 0.04	cmH_2_O s/mL	**0.338**
Tissue resistance	G	3.57 ± 0.62	3.64 ± 0.55	cmH_2_O s/mL	0.807
Tissue elastance	H	20.03 ± 2.24	23.99 ± 1.18	cmH_2_O s/mL	**0.0002**
Hysteresivity	η	0.18 ± 0.03	0.15 ± 0.02	–	**0.025**
Inspiratory capacity	IC	0.91 ± 0.09	0.85 ± 0.07	mL	0.125
Quasi-static elastance	E_st_	12.79 ± 1.38	13.82 ± 1.27	cmH_2_O/mL	0.117
Parameter K of Salazar-Knowles equation	K	0.146 ± 0.005	0.132 ± 0.004	/cmH_2_O	** < 0.0001**
Parameter A of Salazar-Knowles equation	A	0.89 ± 0.08	0.84 ± 0.07	mL	0.163
Total lung capacity	TLC	1.18 ± 0,11	1.07 ± 0.09	mL	**0.035**
Lung volume at 10 cmH_2_O in percentage of TLC	V10_TLC	75 ± 1.11	69 ± 1.48	%	** < 0.0001**
Vital capacity	VC	1.09 ± 0.10	0.96 ± 0.08	mL	**0.008**
Residual volume	RV	0.09 ± 0.02	0.11 ± 0.02	mL	**0.034**
Expiratory reserve volume	ERV	0.22 ± 0.05	0.13 ± 0.04	mL	**0.001**
Functional residual capacity	FRC	0.31 ± 0.05	0.25 ± 0.04	mL	**0.008**
Lung compliance	C	0.07 ± 0.01	0.05 ± 0.01	mL/cmH_2_O	** < 0.0001**
Lung displacement volume	–	0.24 ± 0.04	0.24 ± 0.05	mL	0.792
Resistance (small-amplitude oscillations)	–	0.91 ± 0.06	0.99 ± 0.14	mN s/mm	0.139
Elastance (small-amplitude oscillations)	–	3.67 ± 0.38	4.43 ± 0.77	mN/mm	**0.029**
Hysteresivity (small-amplitude oscillations)	–	0.31 ± 0.02	0.29 ± 0.04	–	0.117
Resistance (large-amplitude oscillations)	–	1.52 ± 0.16	1.69 ± 0.24	mN·s/mm	0.123
Elastance (large-amplitude oscillations)	–	2.89 ± 0.36	3.51 ± 0.71	mN/mm	**0.045**
Hysteresivity (large-amplitude oscillations)	–	0.33 ± 0.02	0.31 ± 0.03	–	0.067

Significant values are in [bold].

**Figure 7:**
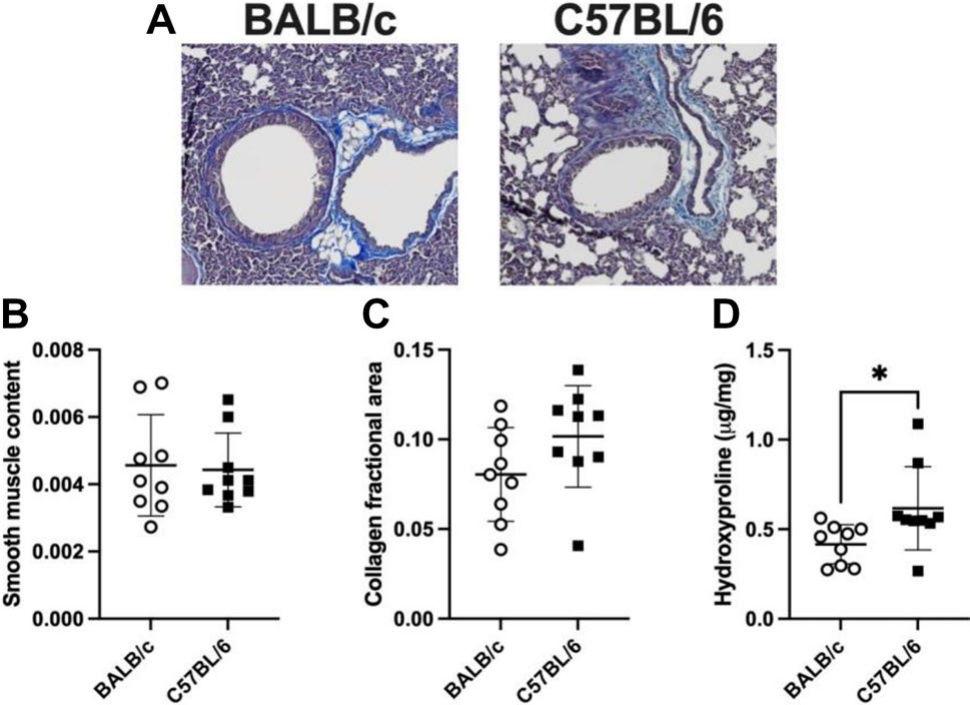
The content of airway smooth muscle and lung collagen. Representative histological images (**A**), histological determinations of the airway smooth muscle content (unitless; because it was calculated for each airway as the area of smooth muscle divided by the square of the basement membrane perimeter) (**B**) and the lung collagen fractional area (**C**), and the determination of the lung hydroxyproline content (**D**) are shown for BALB/c (open circles) and C57BL/6 (solid squares) mice. Data are individual results, together with means ± SD. Significant differences are indicated by asterisks (* is p < 0.05). N = 9 per group.

Histological images and analyses on the right superior and middle lobes are depicted in Fig. 7A–C. The content of airway smooth muscle, expressed in smooth muscle area per basement membrane perimeter square, was not different between the two mouse strains (Fig. 7B). The collagen expression, quantified by measuring the blue fraction of the total lung section stained with Masson trichrome, was numerically but not statistically different between C57BL/6 and BALB/c mice (0.10 ± 0.03 *vs.* 0.08 ± 0.03, p = 0.11) (Fig. 7C). Finally, the content of hydroxyproline in the post-caval lobe is depicted in Fig. 7D. The hydroxyproline content was greater in C57BL/6 than BALB/c mice (0.62 ± 0.23 *vs.* 0.42 ± 0.11, p = 0.03) (Fig. 7D).

## Discussion

This study demonstrated that the lung size, measured by weight and volume displacement of a liquid by the excised lung, are similar between C57BL/6 and BALB/c mice. It also confirmed that the elastance of the respiratory system is greater in C57BL/6 than BALB/c mice when measured in vivo, as testified by increases in respiratory system elastance (E_rs_) and tissue elastance (H), as well as by decreases in the parameter K of Salazar-Knowles equation, lung compliance (C) and the volume at 10 cmH_2_O expressed in percentage of TLC (V_10__TLC). Most volumes measured in vivo were also different between the two mouse strains, being smaller in C57BL/6 than BALB/c mice for TLC, VC, FRC and ERV, and inversely greater in C57BL/6 than BALB/c for RV. Finally, in vitro experiments on isolated lobes demonstrated that tissue elastance was greater in C57BL/6 than BALB/c mice, which was associated with a greater content of hydroxyproline. It is concluded that the lung elastance of C57BL/6 is greater than BALB/c mice mainly because of a stiffer lung tissue due, at least partially, to a greater content of collagen.

Several strains of mice are used to study lung mechanics and for modeling human respiratory diseases. Yet, C57BL/6 and BALB/c mice are the most widely employed^1,2,9^. These two strains exhibit different susceptibilities for the development of specific pathogenic traits reminiscent of human respiratory diseases in response to offending triggers. For example, while C57BL/6 are more prone than BALB/c mice for the development of pulmonary fibrosis upon exposure to bleomycin in models of idiopathic pulmonary fibrosis^2,16^, BALB/c are more prone than C57BL/6 mice for the development of methacholine hyperresponsiveness upon exposure to allergen exposure in models of asthma^9,17^ and for the development of emphysema upon exposure to cigarette smoke or elastase in models of COPD^18,19^. Understanding inherent differences in the mechanical properties of the lung between these two mouse strains may help in interpreting these varying strain susceptibilities. In turn, this may help for guiding the choice of strains in models of human respiratory diseases.

One striking lung difference between C57BL/6 and BALB/c mice is elastance. The elastance of the lung and the respiratory system is greater in C57BL/6 than BALB/c mice^8–14^. This is likely to confer either protection against or vulnerability for the development of specific pathogenic traits. Either way, inferring on the contribution of varying lung elastance to any specific pathogenic trait depends on whether this is due to a smaller lung or a stiffer lung tissue, which has never been delineated. Herein, a comprehensive characterization of lung mechanics was undertaken to determine whether this between-strain difference in lung elastance was due to a different lung volume or to a different stiffness of the lung tissue.

Six readouts were measured to compare respiratory system elastance in vivo between C57BL/6 and BALB/c mice. Importantly, these six readouts are not independent from each other. They are measured using different procedures, but are often sensitive to the same or similar underlying features. Four of them, namely E_rs_, H, E_st_ and C, are sensitive to both lung volume and tissue stiffness. E_rs_ and H, both indicators of elastance, were higher in C57BL/6 *versus* BALB/c mice. Concordantly, C, an indicator of respiratory system compliance, was lower in C57BL/6 *versus* BALB/c mice. E_st_ was also numerically (but not statistically) higher in C57BL/6 than BALB/c mice. Together, these readouts confirmed that the elastance of the respiratory system is greater in C57BL/6 than BALB/c mice. The two additional in vivo readouts include K and V_10__TLC. They are both purportedly insensitive to lung volume^20,21^, and thus exclusively sensitive to lung tissue stiffness. They are currently considered volume-independent indicators of the compliance of the respiratory system^20,21^. They were both significantly lower in C57BL/6 than BALB/c mice. This was the first hint suggesting that the lung elastance was greater in C57BL/6 than BALB/c mice because of a stiffer lung tissue.

In vivo lung volumes were also compared between C57BL/6 and BALB/c mice. TLC, VC, FRC and ERV were all lower in C57BL/6 than BALB/c mice. IC was also numerically (but not statistically) lower in C57BL/6 than BALB/c mice. Inversely, RV was higher in C57BL/6 than BALB/c mice. We reasoned that these bidirectional differences in volume were probably driven by the stiffer lung tissue of C57BL/6 mice. Indeed, a stiffer lung tissue implies that: (1) a smaller lung inflation will be obtained for any given increase in positive pressure, causing lung volumes measured at a positive pressure (such as the 40 cmH_2_O when TLC was measured) to be lower in C57BL/6 than BALB/c mice; and (2) a smaller lung deflation will be obtained for any given increase in negative pressure, causing lung volumes measured at a negative pressure (such as the –10 cmH_2_O when RV was measured) to be higher in C57BL/6 than BALB/c mice. The smaller ERV in C57BL/6 than BALB/c mice actually suggested that less air evacuates the lung from end-tidal expiratory pressure (3 cmH_2_O) to –10 cmH_2_O.

Notably, all in vivo measurements in the present study, including lung volumes, were performed with an intact chest wall. Consequently, the differences in respiratory system elastance between C57BL/6 and BALB/c mice, reported herein and elsewhere^8–12,14^, may also be attributed to between-strain differences in chest wall mechanics. Although possible, Swedin et al.^13^ have demonstrated that lung elastance was also greater in C57BL/6 than BALB/c mice by measuring mechanics in open-chest conditions. This unequivocally confirmed that a greater lung elastance contributes, at least partially and perhaps totally, to the greater respiratory system elastance reported herein and by other^8–12,14^.

In the present study, further in vitro experiments were undertaken to exclude the confounding effect of the chest wall. The techniques employed also exclude confounding effects of all other in vivo factors on lung tissue mechanics, such as circulating or vagally-derived mediators affecting the level of airway smooth muscle activation^20^.

Firstly, the volume of the whole excised lung was measured in vitro by plunging it into Krebs and measuring the displaced liquid volume. The lack of difference between mouse strains confirmed that the lung volume at zero transpulmonary pressure is not different between C57BL/6 and BALB/c mice. This was also consistent with the lack of difference in the lung wet weight between the two mouse strains, as well as the lack of difference in their total body weight.

Secondly, lung tissue mechanics was investigated in vitro on an isolated lobe immerged in Krebs solution. This technique is useful because it directly assesses the mechanics of the lung tissue. The data demonstrated that tissue elastance was greater in C57BL/6 than BALB/c mice, confirming that the lung tissue is stiffer in the former than the latter.

The claim that the lung elastance of C57BL/6 is greater than BALB/c mice because of a stiffer lung tissue and not because of a different lung volume may sound conflicting given that most lung volumes (TLC, VC, FRC, ERV and RV) were significantly different between the two mouse strains. The results could have indeed been misleading if only a high lung volume (*e.g.*, TLC) had been measured without measuring RV and without a complementary set of in vitro data. However, the lack of difference in weight and volume of the excised lung confirmed that the lung size of C57BL/6 and BALB/c mice is similar. The measurement of tissue mechanics on the isolated lobe in vitro also directly demonstrated that the lung tissue of C57BL/6 is stiffer than BALB/c mice. It is with these latter measurements, combined with the measurement of two volume-independent indicators of respiratory system compliance (K and V_10__TLC), that it became clear that the bidirectional difference in volumes observed in vivo (*i.e.*, lower in C57BL/6 than BALB/c mice when measured at positive pressures, while higher in C57BL/6 than BALB/c mice when measured at a negative pressure) were also driven by a difference in lung tissue stiffness. It is, of course, logical that a lung with a stiffer tissue should not only be harder to inflate above zero transpulmonary pressure, but also harder to deflate below zero transpulmonary pressure.

To broadly investigate the factors involved in the increased tissue stiffness of C57BL/6 mice, contents of airway smooth muscle and collagen were measured. The smooth muscle content was not different between C57BL/6 and BALB/c mice. The content of lung collagen was also not statistically different between the two mouse strains when assessed by histology; although a trend was observed for a greater collagen content in C57BL/6 than BALB/c mice. The more precise quantitative assay of hydroxyproline was thus undertaken. It was demonstrated that the content of hydroxyproline is greater in the lung of C57BL/6 than BALB/c mice. These results suggested that the lung of C57BL/6 may be stiffer than BALB/c mice because of a greater content in collagen.

Our finding is important because it was previously demonstrated that stiffening of the lung tissue feeds back adversely to promote further fibrosis and stiffening, particularly by activating lung mesenchymal cells^22,23^. The greater lung tissue stiffness of C57BL/6 mice may thus explain their increased predisposition for the development of fibrosis in various models of respiratory diseases^2,16^, while the lower lung tissue stiffness of BALB/c mice may contribute to their increased predisposition for the development of emphysema^18,19^. This also raises the possibility that the various susceptibilities to suffer from ILD or COPD in humans may rely on the same fundamental elements that govern the inherently differing levels of lung tissue stiffness between C57BL/6 and BALB/c mice. Further mechanistic studies will be required.

### Conclusion

The lung elastance of C57BL/6 is greater than BALB/c mice not because of a smaller lung but because of a stiffer lung tissue due, at least partially, to a greater content of collagen. These new data will be useful for interpreting previous findings and for guiding the choice of mouse strains in experimental models of human respiratory diseases. This study also highlights the importance of conducting a comprehensive analysis of lung mechanics, by using both in vivo and in vitro techniques, to fully understand the underpinnings of any aberration in a specific trait, such as lung elastance. Further studies will still be needed to determine whether other factors than collagen contribute to this differing lung tissue stiffness between C57BL/6 and BALB/c mice, as well as to delineate the molecular mechanisms underlying their differing content of collagen.

## Methods

### Mice

Nine male BALB/c mice (Charles River, Saint-Constant, Canada) and 9 C57BL/6 mice (Jackson, Bar Harbor, MA, USA) were studied at 8–10 weeks of age. They were provided food and water ad libitum at all time. All procedures were approved by the Committee of Animal Care of *Université Laval* following the guidelines from the Canadian Council on Animal Care (protocols 2018-005-4 and 2022-977-1) and comply with the ARRIVE guidelines**.**

### In vivo experiments

#### Respiratory mechanics in mice

Respiratory mechanics were measured with the flexiVent (FX Module 2, SCIREQ, Montreal, QC, Canada) as previously described^24^. Briefly, mice were anesthetized and put under general analgesia using ketamine (100 mg/kg) and xylazine (10 mg/kg). They were then tracheotomized and connected to the flexiVent through an 18-gauge cannula in a supine position. To prevent leakage, a surgical thread was used to secure and seal the trachea on the cannula. They were ventilated mechanically at a tidal volume of 10 mL/kg with an inspiratory-to-expiratory time ratio of 2:3 at a breathing frequency of 150 breaths/min and with a positive end-expiratory pressure of 3 cmH_2_O. Once the ventilation was underway, mice were paralyzed by injecting 100 and 300 µL of pancuronium bromide (0.1 mg/kg) intramuscularly and intraperitoneally, respectively, to avoid spontaneous breathing during the procedure.

Respiratory mechanics were evaluated by probing the lung with two small-amplitude oscillometric perturbations, colloquially called the SnapShot-150 and the Quick Prime-3. The former consists of a single sine wave oscillation at 2.5 Hz that allows the calculation of resistance (R_rs_) and elastance (E_rs_) of the respiratory system based on the linear single-compartment model^25^. The latter is a volume perturbation composed of an input flow signal made of 13 sine waves of mutually prime frequencies with different amplitudes and phases, allowing the impedance of the respiratory system to be calculated from the resulting output pressure signal^26^. The impedance was then analyzed using a computational model called the constant phase model to calculate three parameters^27^. One is Newtonian resistance (R_N_), which reflects the resistance to airflow in conducting airways, although it can sometimes be influenced by the chest wall^28–30^. Another one is tissue resistance (G), which reflects the tissue resistance of the lung and the chest wall^28–30^ but is also sensitive to small airway narrowing heterogeneity^31^. The other one is tissue elastance (H), which reflects the elastance of the whole lung and is thus sensitive to both the accessible (*i.e.*, reachable from the mouth) volume of the lung and the tissue stiffness of the lung and the chest wall^28,29^. The hysteresivity (η), which is the ratio of G over H, was also determined.

#### Lung volumes

Lung volumes were determined as previously described^32,33^. Briefly, three maneuvers of large amplitudes were used: (1) the deep inflation (DI) maneuver; (2) the stepwise, pressure-controlled, partial pressure–volume (P–V) maneuver; and (3) the dynamic, ramp-style, full-range P–V maneuver.

The DI maneuver consists of inflating the lung from 3 to 40 cmH_2_O in 3 s and then maintaining that pressure for another 3 s. The volume that enters the lung from the beginning to the end of the maneuver represents the inspiratory capacity (IC).

The stepwise, pressure-controlled, partial P–V maneuver consists of sequentially inflating the lung through eight steps of increasing pressure (3–40 cmH_2_O) and then deflating it through eight steps of decreasing pressure (40–3 cmH_2_O). Since the pressure is held 1 s after each step, the entire maneuver lasts 16 s. Volume changes at the different holding pressures are recorded and then plotted to form the inflation and deflation limbs of the P–V loop. The descending limb of the P–V loop is then fitted to the Salazar-Knowles equation^34^: V = A−B*e*^−KP^. V and P stand for volume and pressure, respectively, and represent the measured variables. A, B and K are parameters. The A represents the asymptote on the volume axis. It provides an estimate of IC, which, in the present study, was read from the DI maneuver. The parameter B (not shown in the present study) represents the difference between A and the extrapolated volume at which pressure would cross zero. Finally, the K is an exponent describing the curvature of the descending limb. It represents a volume-independent indicator of tissue compliance of the respiratory system^20^. One other readout was extracted from the P–V loop, namely the quasi-static elastance (E_st_) at 5 cmH_2_O. It was calculated from the inverse of the slope of the Salazar-Knowles fit at that pressure.

The dynamic, ramp-style, full-range P–V maneuver has been previously described^33,35^. Briefly, it starts by degassing the lung by ventilating with 100% oxygen for 5 min and then stopping the ventilation for 5 min. During these last 5 min of apnea, oxygen is slowly absorbed, deflating the lung until the alveoli and small airways collapse to achieve a lung volume near zero. A slow constant inflow of air (5 mL/min) is then reintroduced in the degassed lung to inflate it until the pressure reaches 40 cmH_2_O. The flow is then reversed to deflate the lung at the same rate until it reaches – 10 cmH_2_O. The pressure excursion from −10 to 40 mH_2_O is then repeated twice for quality control purposes. Pressure and volume are recorded throughout, such that after the maneuver, three P–V loops can be visualized: one starting from zero volume at zero pressure and two subsequent loops starting from –10 cmH_2_O. The volume entering the lung from a degassed lung to a lung inflated to 40 cmH_2_O is considered the total lung capacity (TLC). The difference in volume between a degassed lung and the volume at –10 cmH_2_O at the end of the deflating limb is considered the residual volume (RV). The difference between TLC and RV is considered the vital capacity (VC). Finally, the difference between TLC and the parameter A of Salazar-Knowles equation from the stepwise, pressure-controlled, partial P–V maneuver is considered the functional residual capacity (FRC), and FRC minus RV is the expiratory reserve volume (ERV)^36^.

Two additional readouts were obtained from the dynamic, ramp-style, full-range P–V maneuver, namely C and V_10__TLC. C represents the compliance of the entire respiratory system. It was calculated as the slope of the linear part of the deflation limb between 3 and 8 cmH_2_O. V_10__TLC is the volume of the lung at 10 cmH_2_O expressed in percentage of TLC. It is used to describe the shape of the deflation limb, and is considered a volume-independent indicator of compliance of the respiratory system^20,21^.

### In vitro experiments

#### Wet weight and physical lung volume at zero transpulmonary pressure

To determine the weight of the wet lung, the entire lung was surgically removed, cleaned, and weighed on a laboratory analytical scale. Then the lung was preserved in cold Krebs solution (111.9 mM NaCl, 5.0 mM KCl, 1.0 mM KH_2_PO_4_, 2.1 mM MgSO_4_, 29.8 mM NaHCO_3_, 11.5 mM glucose, and 2.9 mM CaCl_2_ at a pH of 7.4) until the next analysis. The total lung volume was determined by volume displacement. To this end, the whole lung was immerged into a milliliter-graduated cylinder filled with Krebs solution and the change in volume displaced by the lung was recorded.

#### Lung tissue mechanics

The mechanical properties of the lung tissue were assessed in vitro on the right inferior lobe. The excised lobe was lassoed on both the proximal and the distal extremities with a surgical thread. The distance between the surgical threads was measured. The lobe was then mounted vertically in a 50-mL organ bath. The thread on the distal extremity was attached to a stationary hook and the thread on the proximal extremity was attached to a dual-mode lever arm system (model 300C; Aurora Scientific Inc., Aurora, Canada). The latter not only monitored force but also allowed length excursions (*i.e.*, strains) to be applied. The bath was filled with Krebs solution maintained at 37 °C. The distance between the surgical threads on the lobe was first adjusted to the one measured previously. It was then slightly stretched to an initial distending force of about 3 mN, which slowly settled to about 2 mN. The mechanical properties were measured by subjecting the lobe to length oscillations (Fig. 5). The protocol consisted of a sequence of 2 min of sinusoidal length oscillations with an amplitude of 5% followed by a single larger half-sine stretch of 30% amplitude. All oscillations were imposed at 0.2 Hz. This sequence was repeated 3 times (Fig. 5A).

Analytical tools applicable to both linear and non-linear systems were used to monitor elastance (E), resistance (R), and hysteresivity (η) of the lobe on a cycle-by-cycle basis^37–39^. Briefly, it consists of measuring hysteresis. Hysteresis is the area between the ascending and the descending limbs of the force-strain loop during the cyclical sinusoidal oscillation (Fig. 5C), which is proportional to resistance. Hysteresis is then used to sequentially deduce resistance, the phase angle, η and elastance. The equations are provided here:

- R = 4A/πω(ε^2^), where A is the area of the loop, ω is the angular frequency, and ε is strain.
- ϕ = arcsin ωεR/ΔForce, where ϕ is the phase angle.
- tan ϕ = η
- E = ωR/η

### Histology

Histology was performed as previously described^40,41^ on the right superior and middle lobe. Briefly, the lobes were excised and immersed in formalin during 24 h for fixation. The formalin was replaced by progressively upraising the ethanol concentration to dehydrate the tissue. The lung was then embedded in paraffin and cut transversally in 5 μm-thick sections. Sections were deposited on microscopic slides and stained with Masson trichrome. They were then scanned with a NanoZoomer Digital scanner (Hamamatsu photonics, Bridgewater, NJ, USA) at 40×. To quantify the content of airway smooth muscle, all airways cut transversally in 4 non-contiguous lung sections were analyzed, representing 1–6 airways per mouse (average of 2.8 ± 1.4). The content of airway smooth muscle in each airway was calculated by measuring the area occupied by airway smooth muscle divided by the square of its basement membrane perimeter. A mean was calculated for each mouse and values of all mice within one group were then compiled to obtain a mean per group. To quantify the content of lung collagen, two sections per lobe (ergo four sections per mouse) were used. The entire section of the lung was analyzed. For each section, the blue area was divided by the lung total area. The quantification was done using NDP View Software and ImageJ. A mean from the four sections was calculated for each mouse and values of all mice within one group were then compiled to obtain a mean per group.

### Hydroxyproline

An assay kit was used to quantify the hydroxyproline content (Abcam, Cambridge, United Kingdom). Briefly, the post-caval lobe stored at −80°C was weighed and homogenized with distilled water, in a volume equal to 10 times its weight in μL, using a PowerGen 125 homogenizer (Fisher Scientific, Hampton, NH, USA). 100 μL of the sample subsequently went through alkaline hydrolysis and was heated at 120 °C for 1.75 h. 10 μL of the sample was then transferred in duplicate into the well plate. The absorbance at 560 nm was analyzed using a spectraMax ABS microplate reader (Molecular Devices, San Jose, CA, USA). The results are expressed in μg of hydroxyproline per mg of lung tissue.

### Data analysis

Individual data are presented, together with means ± standard deviations (SD). For all measured readouts, the comparison between mouse strains was evaluated by an unpaired t-test. All statistical analyses were performed with Prism 9 (version 9.1.1, GraphPad, San Diego, CA). Differences with a p ≤ 0.05 were considered statistically significant.

## Data Availability

The datasets used and analyzed during the current study are available from the corresponding author on reasonable request.
